# Alkaptonuria diagnosed in a 4-month-old baby girl: a case report

**DOI:** 10.1186/1757-1626-1-308

**Published:** 2008-11-13

**Authors:** Asok K Datta, Syamali Mandal, Anindya Dasgupta, Tarun K Ghosh

**Affiliations:** 1 Department of Pediatrics Medicine, Burdwan medical College and Hospital, Burdwan, West Bengal, Pin-713101, India; 2 Department of Gynecology and Obstetrics, Burdwan medical College and Hospital, Burdwan, West Bengal, Pin-713101, India; 3 Department of Biochemistry, Burdwan medical College and Hospital, Burdwan, West Bengal, Pin-713101, India; 4 Private practitioner (Pediatrician), Nuripara Lane, Radhanagar, Burdwan-713101, India

## Abstract

The mother of a four month old female baby attended in the well baby clinic with the complaint of black staining of the diaper after few minutes of urination. The baby was born of a non consanguineous marriage, healthy and breast fed. Mother noticed that stain first at the age of two and half month. The urine when kept in a test tube for two hours turned black. Laboratory examination of urine revealed increased concentration of homogentisic acid. The patient was diagnosed as alkaptonuria.

## Introduction

Alkaptonuria (AKU) is a rare metabolic disorder inherited as an autosomal recessive mode. Incidence of this disease is 1 in 250000[1]. There are countries in the world Slovakia for example where this recessive condition is much commoner. Based on a screening programme, highest incidence of AKU (1 in 19000) was recorded in Slovakia [2]. Extensive genealogical studies resulted in the fusion of several 'unrelated' nuclear families into larger pedigrees and enabled tracing most AKU ancestors to their original geographical localities, predominantly in remote mountain areas[2]. The AKU locus was mapped to human chromosome 3q2 by orthology to see the mouse locus aku. Following the cloning of the homogentisate-1,2 dioxygenase (HGD) from human and mouse, different mutations were identified[2]. An analysis of the allelic association with intragenic DNA markers and of the geographic origins of the AKU chromosomes suggests that several independent founders have contributed to the gene pool, and that subsequent genetic isolation is likely to be responsible for the high prevalence of AKU in Slovakia[3].

**Figure 1:**
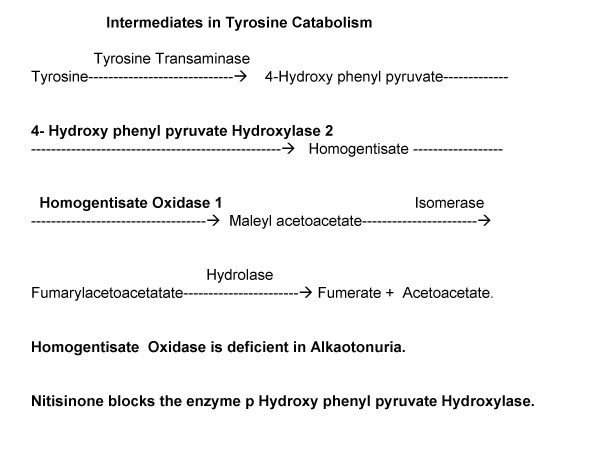
Intermediates in Tyrosine metabolism is shown. Homogentisate oxidase^1 ^which converts thomogentisate to maleyl acetoacetate is deficient in alkaptonuria. 4-Hydroxy phenyl pyruvate Hydroxylase^2 ^can be blocked by the newer drug Nitisinone. In that case there is definite rise of phenylalanine and tyrosine level in blood which is harmful. The drug is not recommended till date for pediatric use.

The disorder is due to deficiency of homogentisate 1,2 dioxygenase also known as homogentisic acid oxidase (HO). HO is required in the metabolism of phenyl alanine and tyrosine during the step when homogentisic acid (HA) is converted to maleyl acetoacetate (Figure 1).

Because of the deficiency of HO, HA is accumulated which is oxidized to benzoquinone acetate which rapidly polymerises. The urine darkens on exposure to air due to oxidation of HA. Late in the disease there is arthritis and connective tissue pigmentation due to the binding of the oxidized polymer [1]. This pigmentation is known as ochronosis.

The ochronotic pigment can be found in the sclera, conjunctiva, limbic cornea, cardiac valve particularly aortic valve, intervertebral disc, muscles and other tissues. Fatal complication may occur in older age[4,5].

## Case presentation

The mother of a four month old female baby attended in well baby clinic with the history of blackish discoloration of diapers after passing urine. She noticed that first at the age of two and half month. The baby was otherwise normal and healthy. She was the first issue, born of a non-consanguineous marriage. The baby was delivered at home. There was no history of antenatal, intranatal and postnatal problem. She was exclusively breast fed and immunized at per.

The family resides in a village named Parijatnagar under Memari police station in the Burdwan district, West Bengal. They are poor in socioeconomic status.

On examination the baby was found alert and active, weight 6 kg, length 64 cm, head circumference 39.5 cm.

Physical and systemic examination revealed no abnormality. White diaper changed to black stain few hours after discharge of urine.

**Figure 2:**
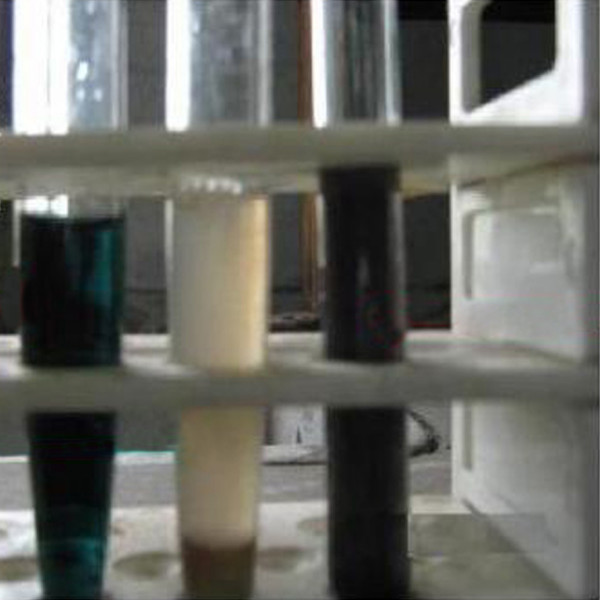
Qualitative assay of homogentisic acid: To 0.5 ml of sample few drops of 10% ammonia was added followed by addition of 3% silver nitrate solution. Development of greenish black color signified presence of substantial amount of homogentisic acid. The test tube in the centre is the control one.

Qualitative urine examination showed dark greenish black discoloration due to presence of homogentisic acid (Figure 2). Quantitative examination of urine revealed concentration of homogentisic acid in urine was 112 mg/dl (normally HA is not present in urine). Examination of eyes, musculoskeletal systems, skin, and cardiovascular system was normal.

As there is no definite treatment recommendation at this age the mother was advised to continue breast feeding and to attend after six months of age for follow up.

## Discussion

Alkaptonuria is a very rare disorder. The urine turns black on standing or staining the diaper is the first feature of this disorder but majority of parents fail to note or attend with this complaint rather an adult patient with this disorder usually present in the fourth decade with arthritis, ocular, cutaneous and cardiovascular ochronosis [4,5]. There are reports of aortic valve stenosis and concomitant coronary artery disease [6]. A case of bilateral spontaneous rupture of both quadriceps tendons has been described [7].

So far we have reviewed the literature our case is the earliest age of presentation from India. Another early presentation was reported at the age of ten years with the complaint of bluish discoloration of sclera [8].

There is no definite treatment of this disorder. Dietary restriction of protein is not recommended in children. Vitamin C supplementation as an antioxidant is not helpful.

Nitisinone, a tyrosine degradation inhibitor, has been very restricted use in experimental treatment. It inhibits 4-hydroxyphenylpyruvate hydroxylase, which mediates formation of homogentisic acid (Figure 1). It may prevent ochronosis [1]. There is no recommendation for pediatric use till date.

## Conclusion

In Alkaptonuria various new mutations are reported indicating that most mutations are unique to a family. Our patient is probably a case of fresh mutation.

The patient is diagnosed very early. The parents are informed about the disease and the need for further follow up of the case so that early detection of complication in later age and adequate management is possible.

## Parent's perspective

We have noticed black discoloration of napkins after toilet and urine was black when kept in glass bottle. We went to the doctor and were advised for urine test and other investigation. Now as our baby is breast fed, so doctor advised us to check after six months for weaning advises.

## Consent

Written informed consent was obtained from the patient for publication of this case report and accompanying images. A copy of the written consent is available for review by the Editor-in-chief of this journal.

## Competing interests

The authors declare that they have no competing interests.

## Authors' contributions

AKD analyzed and interpreted the patient data regarding the disease. SM is a major contributor in writing the manuscript. TKG first examined the patient and referred to AKD, TKG helps in follow up of the case regularly. AD helped in biochemical examinations.
